# The role of sickness absence diagnosis for the risk of future inpatient- or specialized outpatient care in a Swedish population-based twin sample

**DOI:** 10.1186/s12889-021-10942-2

**Published:** 2021-05-20

**Authors:** Annina Ropponen, Mo Wang, Jurgita Narusyte, Sanna Krkkinen, Victoria Blom, Pia Svedberg

**Affiliations:** 1 Division of Insurance Medicine, Department of Clinical Neuroscience, Karolinska Institutet, SE-171 77 Stockholm, Sweden; 2 Finnish Institute of Occupational Health, Helsinki, Finland; 3 Center of Epidemiology and Community Medicine, Stockholm County Council, Stockholm, Sweden; 4 The Swedish School of Sport and Health Sciences, Stockholm, Sweden

**Keywords:** Longitudinal, Health care, Sick leave, Diagnosis, ICD-10, Population-based, Twins

## Abstract

**Background:**

Studies of consequences of sickness absence such as health and well-being have been rare whereas risk factors for sickness absence have been studied extensively. This study assumed the consequences of sickness absence would differ by diagnostic group or by patient care type. The aim was to investigate sickness absence due to various diagnosis groups as a predictor for subsequent inpatient- and specialized outpatient care while controlling for familial confounding.

**Methods:**

We utilized the register data of 69,552 twin individuals between 16 and 80years of age (48% women). The first incident sickness absence spell, from baseline year 2005, including diagnosis of sickness absence was our primary exposure of interest and we followed them until the first incident inpatient- and specialized outpatient care episode with main diagnosis code or until 31.12.2013.

**Results:**

A total of 7464 incident sickness absence spells took place (11%), 42% had inpatient care and 83% specialized outpatient care (mean follow-up time 3.2years, SD 3.1years). All the main sickness absence diagnosis groups were associated with increased risk of future care in comparison to no sickness absence. Controlling for confounders attenuated the associations in magnitude but with retaining direction, and we could not confirm an effect of familial factors.

**Conclusions:**

Sickness absence predicts both inpatient- and specialized outpatient care and the association is universal across diagnosis groups. The lower survival time and incidence rates of inpatient than specialized outpatient care point towards severity of diseases assumption. This finding was also universal across sickness absence diagnosis groups.

**Supplementary Information:**

The online version contains supplementary material available at 10.1186/s12889-021-10942-2.

## Background

Reducing the extent of work incapacity in terms of sickness absence (SA) is highly prioritized in welfare countries. The incidence of SA is high and has also increased lately, even before the current pandemic of Covid-19, despite improved health conditions in European countries [1, 2]. Hence, absence from the labor market due to SA is a public health concern in several European countries [2]. A large amount of studies have previously reported associations between individual sociodemographic, socioeconomic, health- and work-related factors and future SA [3,6]. However, far less studies have investigated the consequences of SA, although such are considerable both for the individual, employers and for society. For example SA is linked with a number of negative health-related consequences, such as disease (the same as being granted SA for, or another), worse well-being, weaker economy, less career development, worse social integration, and premature death [7]. Since SA is a mean to allow an individual to recover and retain work capacity, studies to investigate the consequences of various SA causes (i.e. diagnoses) have emerged.

SA is commonly prescribed in healthcare [8]. A lot of research have indicated that SA plays a role for many consequences, not only for disability pension (DP) [9, 10], but also for mortality or even suicide [11,13]. However, even theoretically the effects of SA for an individual are complex and varying based on the life situation [14, 15], whereas SA is also known to increase the risk of reoccurring SA, DP and unemployment [16,19]. On the other hand, besides SA, also patient care in terms of inpatient- or specialized outpatient care, is often needed for various symptoms and diseases [20, 21]. Some studies have addressed SA as a predictor of patient care [15, 22] although usually the pathway has been estimated utilizing the initiation from care, i.e. from onset of a disease, symptom or medication that required medical attention in a care unit, to SA [23, 24]. Therefore, a need exists to explore the consequences of SA at diagnosis group level to understand the process of SA [15].

Further, many chronic diseases, which also are grounds for SA, have moderate to large genetic influence. For example, genetics is known to play a role in low back pain [25], depression [26], anxiety [27], and e.g. blood pressure [28]. Since genetics is also known to affect SA [9, 22], there is a need for studies accounting for familial factors (genetics and mainly childhood environment) in associations of SA and potential consequences. A twin study with a co-twin control design utilizing twin pairs discordant for a factor of interest provides possibility to define if an association between SA and inpatient care is influenced by familial factors [29, 30].

In this study, we hypothesized that associations between SA due to a diagnostic group and patient care would follow broadly the severity of disease assumption [31] i.e. operationalized in this study by register data of diagnoses and inpatient vs. specialized outpatient care. Hence, we aimed to investigate if SA due to various diagnosis groups differ in the associations with subsequent inpatient- and specialized outpatient care. In specific, we aimed to control for the effect of familial confounding (genetics and early, shared environment) utilizing co-twin control design in the associations.

## Materials and methods

This study utilized the register data of the Swedish Twin project Of Disability pension and Sickness absence (STODS) [32] with all 119,907 twins from the Swedish Twin Registry (STR) born 19251990 [33]. One-third of the twins are monozygotic (MZ) and one-third same-sexed dizygotic (DZ) and last proportion opposite-sexed DZ twins [33]. For this study, the sample was limited to those 16years of age, alive, living in Sweden, not on DP, or inpatient- or specialized outpatient care in 2005, and followed from the Micro-Data for Analyses of Social Insurance (MiDAS) database from the National Social Insurance Agency. We used the unique ten-digit Swedish identification number for the linkage of data from the national registers.

All residents in Sweden are eligible for the national SA system if they are 16years of age, and have income from work, or unemployment or student benefits if they have a disease or injury causing work incapacity. All residents are also entitled to patient care [34].

Hence for the study period from 2005 until the end of 2013, we included dates and diagnoses for all SA (>2weeks) reimbursed by the Swedish Social Insurance Agency, in- and specialized outpatient care registries for dates and diagnoses from the National Board of Health and Welfare, and date of deaths from the causes of death register (for censoring). We could not control the SA before 2005. Emigration (date) was included as another censoring reason (from Statistics Sweden the Longitudinal Integration Database for Health Insurance and Labor Market Studies Register [LISA by Swedish acronym]) [35].

**Fig. 1:**
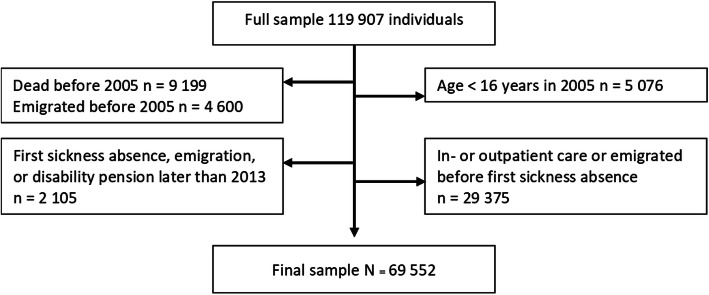
Flow chart of the study sample

The final sample consisted of 69,552 twin individuals (Fig.1). The number of complete twin pairs was 5416 MZ, 6757 DZ same-sexed, and 3251 DZ opposite-sexed twin pairs.

Those with first SA, emigration, or disability pension later than 2013 were excluded to avoid reversed causality, i.e. appearance of these shortly after the end of the follow-up. The final sample included censoring for International Classification of Diseases 10th Revision (ICD-10) codes O00-O99: Pregnancy, childbirth and the puerperium. Pregnancy and childbirth were not considered as illnesses in this study and since many might have SA or in- or outpatient care during that time, we did not account them in the analyses, but estimated them as censored. The number of SA due to O00-O99 was 256, inpatient care due to O00-O99 was 3109 and specialized outpatient care due to O00-O99 was 467.

### Diagnosis of a sickness absence spell

We used the first incident SA spell for the diagnosis of SA as our primary exposure of interest during the follow-up from 2005 until the end of 2013. First, we reported all the SA diagnosis groups at ICD-10 main rubric level (Supplemental Table 1), but also categorized the SA spell diagnoses into five categories based on the frequencies in the final sample: F00-F99 (Mental and behavioural disorders), M00-M99 (Diseases of musculoskeletal system), J00-J99 (Diseases of respiratory system), all other diagnoses and no SA (Table 1). Since the data also included SA without diagnosis (missing information in the database), we included them as an own category.

**Table 1:** Descriptive characteristics across inpatient and specialized outpatient care vs. no care (the inpatient and specialized outpatient care are not mutually exclusive) among those with SA

Categorized SA diagnosis	Inpatient care (***n***=25,947)		Outpatient care (***n***=54,985))		No care (***n***=13,942))	
	n	%	n	%	n	%
No SA	23,422	90	49,377	90	12,190	87
F00-F99: Mental and behavioural disorders	542	2	1325	2	459	3
M00-M99: Diseases of musculoskeletal system	687	3	1554	3	359	3
J00-J99: Diseases of respiratory systems	210	1	440	1	116	1
any other diagnosis	762	3	1529	3	501	4
missing diagnosis	324	1	760	1	317	2
Sex (women)	12,805	49	25,989	47	7108	51
Education						
Low ( 9years)	8036	31	14,387	26	2646	19
Intermediate (1012years)	9129	35	22,244	40	6361	46
High ( 13years)	4426	17	13,096	24	4672	34
Family situation						
Married or cohabitant without children	11,199	43	19,024	35	2115	15
Married or cohabitant with children	2139	8	8471	15	3800	27
Single without children	12,177	47	26,286	48	7566	54
Single with children	428	2	1189	2	450	3
Type of living area						
Big cities	7736	30	18,159	33	4624	33
Medium-sized cities	9353	36	19,759	36	5076	36
Rural areas	8854	34	17,052	31	4231	30
	**Mean**	**SD**	**Mean**	**SD**	**Mean**	**SD**
Age (at baseline)	59.0	16.0	51.2	18.0	38.1	14.5

### Inpatient- and specialized outpatient care episode

The first incident inpatient- and specialized outpatient care episode with main diagnosis code (ICD-10) after first incident SA spell were our study outcomes. Inpatient care is hospitalization (typically provided in hospitals) of any length, whereas specialized outpatient care includes home care and visits to the specialized care according to Swedish health care setting [36]. The follow-up was from 1.1.2005 until 31.12.2013 and the censoring was date of emigration or death, whichever occurred first.

### Covariates

This study used age, sex, family situation (i.e. a combination of marital status and children living at home), education (i.e. level of education categorized into: low (<9years of education), intermediate (1012years of education), and high (>13years of education)), and type of living area which are so-called homogenous regions (H-regions) [37] grouped municipalities into three categories of big cities, medium sized cities, and rural areas from Statistics Sweden the LISA database in 2005 [35] as covariates based on the known association both with SA [38] and study outcomes [39].

### Statistical analyses

We described the sample using frequencies and proportions. Cox proportional hazards regression models were estimated for hazard ratios (HR) with 95% confidence intervals (CI) using two outcomes: first incident inpatient care or specialized outpatient care. The models for the whole final sample were adjusted for age and sex while accounting the non-independency within twin pairs by clustering for 95% CIs. The covariates (education, family status and living area) were included all at the same time to the model (i.e. full model). We ran the models with two strategies: first with those without SA (no SA) as reference group for the overall effect of SA on study outcomes and second with those with SA but missing diagnosis as reference group to clarify further the roles of diagnoses on the associations. The assumption was that if the diagnosis in sickness absence would be of importance, then results with no sickness absence vs. sickness absence with missing diagnosis would differ. Furthermore, we present HR with 95%CI for all ICD-10 main categories with study outcomes in the Supplemental Table 3, but these results are not used for interpretations.

Conditional Cox proportional hazards regression models for discordant twin pairs were estimated to investigate the potential confounding by familial factors (i.e. genetics and early shared environment). The conditional Cox models include only same-sex twin pairs discordant for study outcomes which means that within a pair, a twin had a patient care episode while the co-twin had not. Through conditionality, each twin pair has their own baseline hazard and hence control for familial confounding. The interpretation of discordant pair analyses comes from the comparison of the results to the models of the whole cohort. If familial confounding affects the results, the results based on the analyses of the whole cohort are not confirmed in the conditional models. Vice versa, lack of familial confounding shows if the results of the whole cohort are also found within discordant twin pairs (i.e. conditional models).

To test the proportionality of hazards, we estimated Kaplan-Meier survival curves across SA diagnosis groups to assess their differences but also utilized log-rank tests to analyze survival differences. The person-time-at-risk, incidence rate and 25% quartiles for survival time were also estimated. The statistics were analyzed with Stata version 14.2 MP (Stata Corporation, College Station, TX, USA).

### Ethical approval

This study protocol was designed and performed according to the principles of the Helsinki Declaration. The study was approved by the Regional Ethical Review Board in Stockholm.

## Results

In the final sample, mean age at baseline in 2005 was 48.6years (range 1680, SD 18.2) and 48% were women. Since baseline, 7464 first incident SA spells took place (11% of the final sample) and in the final sample, 42% had inpatient care and 83% specialized outpatient care. The mean follow-up time was 3.2years (range 09.0, SD 3.1years).

The main diagnoses for inpatient care were I00-I99 (*n*=4394), R00-R99 (*n*=3042), and S00-T98 (*n*=2954), and for outpatient care ICD-10 Z00-Z99 (*n*=6980), S00-T98 (*n*=5140), and M00-M99 (*n*=4543) (Supplemental Table 2). Comparison of inpatient-, outpatient- and no care groups indicated that those with care were older, more often less educated and likely married without children than those without patient care (Table 1).

**Fig. 2:**
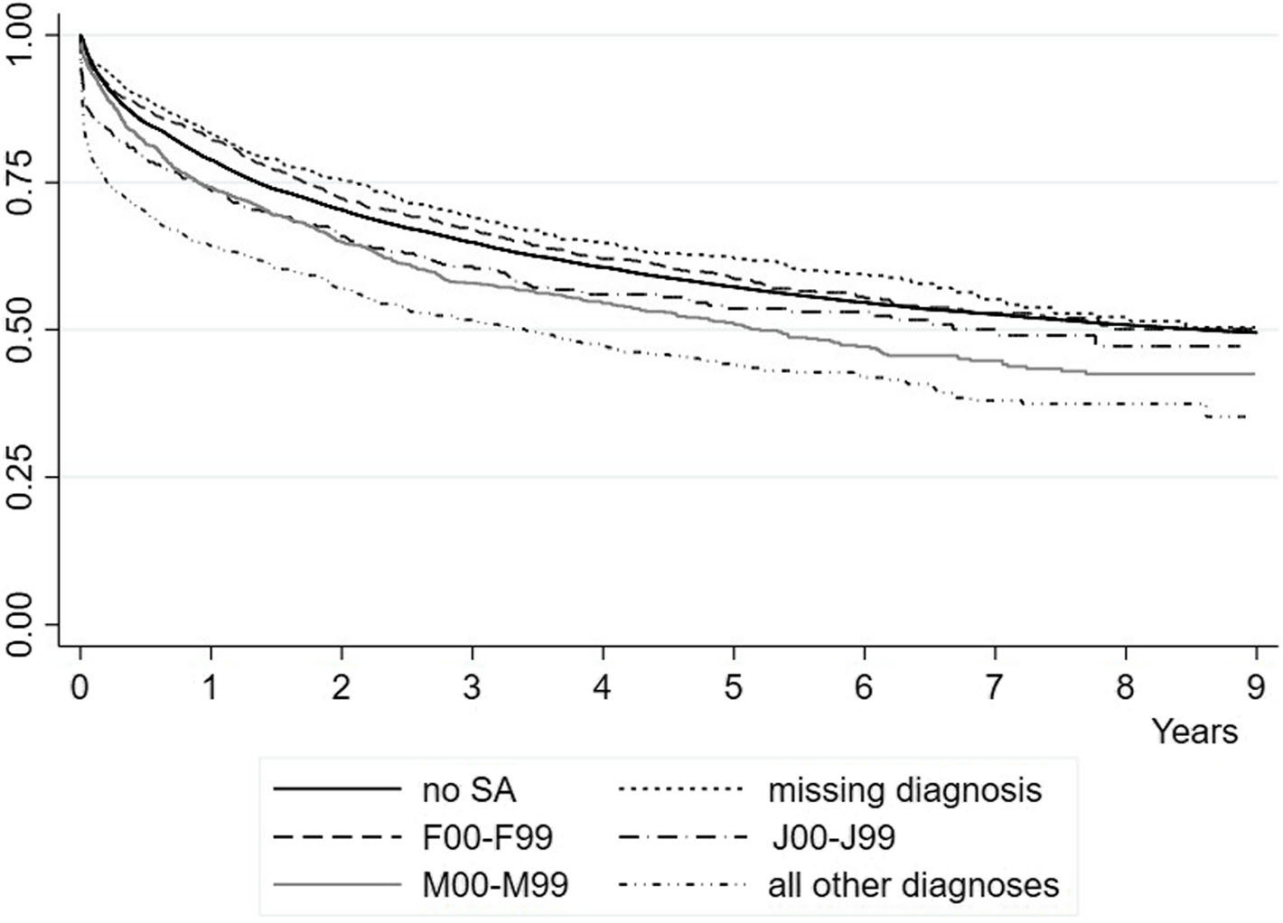
Kaplan-Meier Survival curves across SA diagnosis categories for inpatient care (log-rank test for equality of survivor functions *p*<0.001)

**Fig. 3:**
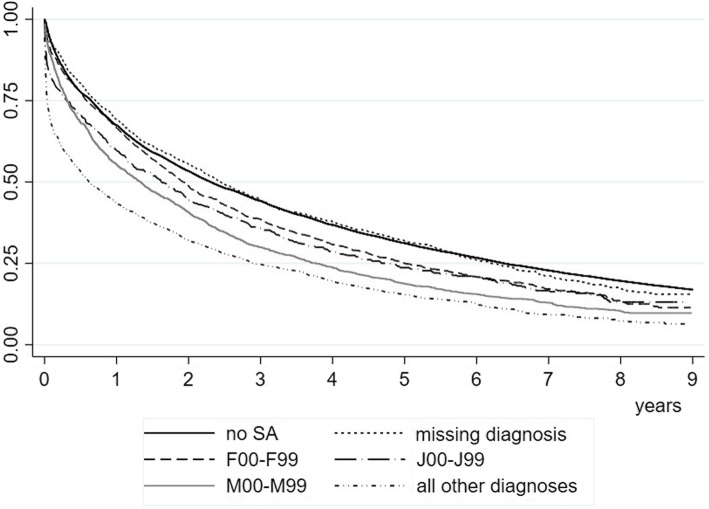
Kaplan-Meier Survival curves across SA diagnosis categories for specialized outpatient care (log-rank test for equality of survivor functions *p*<0.001)

Figure2 presents the Kaplan-Meyer survival curves for inpatient care across the SA diagnoses categories and Fig.3 for specialized outpatient care showing similar patterns for the risk of patient care across the main diagnosis categories of SA over the years of follow-up.

The main SA diagnosis groups (F00-F99, J00-J99, M00-M99, any other diagnoses and missing diagnoses were all strongly associated with increased risk of future care (Table 2) in comparison to no SA. Instead, in comparison to those with missing diagnosis for SA, no SA, SA due to M00-M99, and SA due to any other diagnosis were associated with increased risk of both inpatient and specialized outpatient care (whereas SA due to J00-J99 only with inpatient care). Controlling for confounders (family situation, living area, and education) attenuated the associations in magnitude with retained direction.

**Table 2:** Cox proportional hazards regressions (HR) with 95% confidence intervals (CI) for associations between SA diagnosis categories and in- or outpatient health care (SA due to O00-O99: Pregnancy, childbirth and the puerperium excluded). The upper rows of the table utilized no SA as reference category, whereas the lower part below the middle line had missing diagnosis as reference

Categorized SA diagnosis	Inpatient care				Outpatient care			
	Age and sex adj		Full model^a^		Age and sex adj		Full model^a^	
	HR	95%CI	HR	95%CI	HR	95%CI	HR	95%CI
No SA	1	ref	1	ref	1	ref	1	ref
F00-F99: Mental and behavioural disorders	**2.27**	**2.01, 2.56**	**1.30**	**1.19, 1.42**	**1.61**	**1.49, 1.73**	**1.61**	**1.49, 1.73**
M00-M99: Diseases of musculoskeletal system	**2.37**	**2.08, 2.71**	**1.49**	**1.37, 1.61**	**1.83**	**1.68, 1.98**	**1.80**	**1.66, 1.96**
J00-J99: Diseases of respiratory system	**2.83**	**2.23, 3.59**	**1.50**	**1.29, 1.74**	**1.80**	**1.53, 2.10**	**1.81**	**1.54, 2.12**
Any other diagnosis	**4.22**	**3.72, 4.79**	**2.34**	**2.15, 2.55**	**2.71**	**2.47, 2.97**	**2.71**	**2.47, 2.98**
Missing diagnosis	**1.91**	**1.64, 2.22**	**1.19**	**1.07, 1.33**	**1.37**	**1.25, 1.50**	**1.37**	**1.25, 1.50**
No SA	**0.52**	**0.45, 0.61**	**0.84**	**0.75, 0.94**	**0.67**	**0.62, 0.73**	**0.66**	**0.61, 0.72**
F00-F99: Mental and behavioural disorders	1.19	0.99, 1.44	1.09	0.95, 1.26	1.01	0.91, 1.13	1.00	0.90, 1.11
M00-M99: Diseases of musculoskeletal system	**1.24**	**1.02, 1.51**	**1.25**	**1.09, 1.43**	**1.17**	**1.05, 1.31**	**1.18**	**1.05, 1.31**
J00-J99: Diseases of respiratory system	**1.49**	**1.12, 1.97**	**1.26**	**1.04, 1.51**	1.09	0.91, 1.29	1.10	0.92, 1.31
Any other diagnosis	**2.21**	**1.83, 2.68**	**1.97**	**1.71, 2.26**	**2.14**	**1.91, 2.41**	**2.15**	**1.91, 2.41**
Missing diagnosis	1	ref	1	ref	1	ref	1	ref

^a^ Adjusted for age, sex, family situation, living area, and education

Assessment of results of discordant twin pairs (Table 3) shows weak or no effect of familial confounding since the HR were in the same direction and only slightly attenuated among the DZ pairs. Table4 summarizes the incidence rates and time at risk for the SA main diagnosis groups showing a longer survival time for specialized outpatient care than inpatient care.

**Table 3:** Conditional Cox proportional hazards regressions (HR) with 95% confidence intervals (CI) for associations between SA diagnosis categories and in- or outpatient health care

Categorized SA diagnosis	Inpatient care						Outpatient care					
	All discordant pairs (***n***=7421)		MZ (***n***=1591)		DZ (***n***=2380)		All discordant pairs (***n***=5189)		MZ (***n***=1218)		DZ (***n***=1500)	
	HR	95%CI	HR	95%CI	HR	95%CI	HR	95%CI	HR	95%CI	HR	95%CI
No SA	1	ref	1	ref	**1**	**ref**	**1**	**ref**	1	ref	**1**	**ref**
F00-F99: Mental and behavioural disorders	1.32	0.96, 1.81	**1.75**	**1.07, 2.87**	1.09	0.72, 1.66	**1.40**	**1.13, 1.73**	**1.56**	**1.13, 2.16**	**1.29**	**0.97, 1.72**
M00-M99: Diseases of musculoskeletal system	**2.01**	**1.51, 2.66**	**2.01**	**1.27, 3.19**	**2.03**	**1.41, 2.90**	**1.84**	**1.52, 2.22**	**1.76**	**1.31, 2.37**	**1.92**	**1.50, 2.46**
J00-J99: Diseases of respiratory system	**1.93**	**1.19, 3.13**	2.13	0.99, 4.55	1.78	0.96, 3.33	**1.79**	**1.28, 2.50**	**2.46**	**1.44, 4.21**	1.43	0.93, 2.20
Any other diagnosis	**4.55**	**3.27, 6.32**	**7.47**	**4.12, 13.55**	**3.46**	**2.32, 5.17**	**3.73**	**2.98, 4.66**	**4.94**	**3.42, 7.13**	**3.06**	**2.31, 4.07**
Missing diagnosis	**1.75**	**1.17, 2.61**	**1.93**	**1.02, 3.65**	1.63	0.96, 2.75	**1.33**	**1.03, 1.72**	**1.63**	**1.10, 2.42**	1.13	0.81, 1.59

**Table 4:** Summary of time at risk and incidence rates with quartiles of survival time for inpatient and specialized outpatient care across SA spell duration categories

Categorized SA diagnosis	time at risk (person years)	number of subjects	Inpatient care				Outpatient care			
			incidence rate	Survival time			incidence rate	Survival time		
				25%	50%	75%		25%	50%	75%
No SA	207,505.31	62,077	0.11	1.36	8.57		0.24	0.65	2.31	6.38
F00-F99: Mental and behavioural disorders	4367.05	1804	0.12	1.72			0.30	0.58	1.92	5.00
M00-M99: Diseases of musculoskeletal system	4023.93	1923	0.17	0.90	5.20		0.39	0.31	1.33	3.78
J00-J99: Diseases of respiratory system	1346.18	565	0.16	0.87	7.01		0.33	0.30	1.62	4.80
Any other diagnosis	2742.25	2048	0.28	0.21	3.40		0.56	0.04	0.62	2.89
Missing diagnosis	3019.18	1088	0.11	2.11			0.25	0.71	2.43	6.23
Total for those with SA	223,003.90	69,505	0.12	1.32	8.26		0.25	0.60	2.20	6.20

-=no observations

## Discussion

This study with 69,552 twin individuals was designed to investigate if SA due to various diagnosis groups differ in the associations with for subsequent inpatient- and specialized outpatient care while controlling for the effect of familial confounding (genetics and early, shared environment). Our results indicate that the main SA diagnosis (F00-F99, J00-J99, M00-M99, any other diagnoses and missing diagnoses) were all associated with increased risk of future hospital care and the association was slightly affected by the confounding factors including familial ones but more among inpatient than in outpatient specialized care. Although SA is common prescription in healthcare [8], the studies of the consequences of SA have been rare. Therefore our study adds to the literature about the link between SA and a number of negative health-related consequences (disease whether or not the same as being granted SA for, or another) [7, 15]. Furthermore, we addressed the consequences of SA at specific, i.e. diagnosis group level to understand the process of SA [15].

Another part of our results focused on testing the group missing SA diagnosis as a reference instead of no SA group. Those results indicated a clear protective role of not having SA for inpatient or specialized outpatient care. Furthermore, SA due to M00-M99 and SA due to any other diagnosis were associated with both inpatient- and specialized outpatient care. However, our results for other SA diagnosis groups maintained the direction towards higher risk, being indicative that the effect is universal across the diagnosis groups. Taken together, these results do not fully confirm our hypothesis that the associations between SA and patient care would differ by diagnoses or by patient care type i.e. inpatient vs. specialized outpatient care. Instead our results support the theoretical assumption that consequences of SA should be investigated at specific, i.e. diagnosis group level to understand the process of SA [15].

Yet we cannot completely reject our hypothesis. We detected higher incidence rates and survival times for specialized outpatient care than for inpatient care. This might indicate a trend towards severity of disease assumption, i.e. that patient care type plays a role. However, since patient care in terms of inpatient- or specialized outpatient care is often needed for various symptoms and diseases [20, 21], further research is needed to clarify the severity of diseases assumption with more detailed investigation of diagnoses within main ICD-rubrics and also for this association. One may speculate that organization of health care may play a role among those with SA, alternatively self-medication or other self-help may influence the decision of seeking care after SA (towards less contact to health care), stigma related to e.g. mental health symptoms (also towards less contact to health care) or there may be some other influential factors [40,43]. These factors could not be controlled in this study, but if existing, they might have diluted the results.

The utilization of a twin sample enabled us to control for familial confounding. This adds to epidemiological knowledge based on the individuals in which no genetics or shared (usually early childhood) environmental factors can be controlled for [29, 30]. Our results based on the comparison of the whole cohort to discordant twin pairs showed the point estimates to retain the magnitude and direction, suggesting there is weak or no effect of familial confounding in the associations between SA due to various diagnosis groups and patient care. Hence this independency from familial confounding suggest direct link between SA and patient care although we expected to see familial confounding based on the genetic influence on many chronic diseases that leads to SA [25,28], but also on SA itself [9, 22]. For (occupational) health care including any preventive actions for SA and/or hospitalizations in terms of inpatient- or specialized outpatient care means both tailored interventions specified by the underlying condition or diagnosis as well as monitoring SA. This arises from the fact that our results point toward an assumption that SA might be an independent and early indicator (i.e. due to weak or no effects of confounders including familial ones) for the need of care. Besides being a common prescription in health care and a part of treatment processes, SA could be utilized as a preventive means for need of care, i.e. being indicative for workplace or individual level actions. These actions could be part of communication based on incidence of SA between health care providers, workplaces and communities for relevant care or prevention.

This longitudinal, population-based register study of Swedish twins has several strengths. The register data is comprehensive, of high quality and without bias or loss due to drop out. We had the opportunity to explore all the main diagnosis groups of ICD-10 both for SA and hospitalizations adding precision. Furthermore, we also tested both no SA and those with SA but missing diagnosis as reference groups to assess the importance of diagnosis which has rarely been done before. However, this kind of register study has also some pitfalls. One limitation was the fact that we only had diagnosis for SA since 2005 limiting the length of the follow-up up to maximal 9 years. Hence one may assume that even longer follow-up might add knowledge on the associations. Another limitation is based on the diagnosis groups. One may assume a tendency of e.g. severe psychiatric disorders to be treated in inpatient care whereas common mental disorders might be addressed in primary health care. This might bias our results due to the underlying disease leading the treatment decisions and potentially being linked with our severity of diseases assumption. Hence even more comprehensive studies with access to high quality register data of primary care and health care level (i.e. primary, occupational, inpatient or outpatient care) of issuing SA for full coverage of all types of care would be needed in the future. The existing register data allowed us to control for several factors in the analyses (i.e. age, sex, education, family status and living area besides genetics and family background). However, we could not control SA before baseline in 2005 which may have overestimated the association since earlier SA are known to predict later SA [44]. Since we utilized education as a proxy for socioeconomic status including employment, we did not account the employment status which could be controlled in further studies. In addition, due to the process in acquiring SA and then seeking patient care, there might be other influential factors related to living situation, working life, personality or else that should be collected via surveys or other means and therefore not available for us. Consequently, we may have overestimated the associations and further studies should confirm if this was the case. The Swedish sample with register data from a welfare country with SA benefits and patient care for all citizens [36] can limit the generalizability of our results. We though assume that these findings can be useful within Nordic countries with similar welfare status and indicative also for other countries for adding to the knowledge for tailoring interventions for underlying conditions or diagnosis.

## Conclusions

Sickness absence predicts both inpatient and specialized outpatient care and these associations were universal across diagnosis groups. On the other hand, the survival time and incidence rates of inpatient care were lower than for specialized outpatient care pointing towards the assumption of severity of diseases although this finding was universal across diagnosis groups of sickness absence.

## Supplementary Information

**Additional file 1: Supplemental Table 1.** Frequencies of ICD-10 main diagnosis categories for SA among those with inpatient or specialized outpatient care during the follow-up. **Supplemental Table 2.** Frequencies of diagnoses of inpatient and specialized outpatient care episodes among individuals with and without sickness absence (SA). **Supplemental Table 3.** Cox proportional hazards regressions (HR) with 95% confidence intervals (CI) for associations between SA diagnoses at ICD-10 main diagnosis categories and in- or outpatient health care.

## Data Availability

The datasets generated and analysed during the current study are not publicly available. According to the General Data Protection Regulation, The Swedish law SFS 2018:218, The Swedish Data Protection Act, the Swedish Ethical Review Act, and the Public Access to Information and Secrecy Act, these type of sensitive data can only be made available after legal review, for researchers who meet the criteria for access to this type of sensitive and confidential data. Readers may contact the last author regarding these details.

## Abbreviations

CI: Confidence interval
DP: Disability pension
DZ: Dizygotic
HR: Hazard ratio
ICD: International Classification of Diseases 10th Revision
LISA: Longitudinal Database for Health Insurance and Labor Market Studies Register
MiDAS: Micro data for Analyses of Social Insurance
MZ: Monozygotic
SA: Sickness absence
SD: Standard deviation
STODS: Swedish Twin project Of Disability pension and Sickness absence
STR: Swedish Twin Registry
