# Supplementary material for: The role of sickness absence diagnosis for the risk of future inpatient- or specialized outpatient care in a Swedish population-based twin sample
Source: BMC Public Health. 2021 May 20;21:957. doi: 10.1186/s12889-021-10942-2 (PMC8136267; doi:10.1186/s12889-021-10942-2)
Supplement: Supplementary file 1 — Additional file 1: Supplemental Table 1. Frequencies of ICD-10 main diagnosis categories for SA among those with inpatient or specialized outpatient care during the follow-up. Supplemental Table 2. Frequencies of diagnoses of inpatient and specialized outpatient care episodes among individuals with and without sickness absence (SA). Supplemental Table 3. Cox proportional hazards regressions (HR) with 95% confidence intervals (CI) for associations between SA diagnoses at ICD-10 main diagnosis categories and in- or outpatient health care. [file 12889_2021_10942_MOESM1_ESM.docx]

**The role of sickness absence diagnosis for the risk of future inpatient- or specialized outpatient care in a Swedish population-based twin sample**

**Authors:** Annina Ropponen^1,2^, Mo Wang^1^, Jurgita Narusyte^1,3^, Sanna Kärkkäinen^1^, Victoria Blom^1,4^, Pia Svedberg^1^

| ICD-10 main categories for SA | Inpatient care | Outpatient care | No care |
| --- | --- | --- | --- |
|  | n | n | n |
| A0-A99, B0-B99: Certain infectious and parasitic diseased | 60 | 114 | 29 |
| C00-D48, Neoplasms | 28 | 33 | - |
| D50-D89: Diseases of the blood and blood-forming organs | 12 | 26 | na |
| E00-E90: Endocrine, nutritional and metabolic diseases | 28 | 40 | na |
| F00-F99: Mental and behavioural disorders | 542 | 1325 | 459 |
| G00-G99: Diseases of the nervous system | 41 | 99 | 14 |
| H00-H59: Diseases of the eye and adnexa | na | 13 | na |
| H60-H95: Diseases of the ear and mastoid process | 19 | 37 | na |
| I00-I99: Diseases of the circulatory system | 93 | 151 | 23 |
| J00-J99: Diseases of the respiratory system | 210 | 440 | 116 |
| K00-K93: Diseases of the digestive system | 76 | 123 | na |
| L00-L99: Diseases of the skin and subcutaneus tissue | 21 | 59 | 13 |
| M00-M99: Diseases of the musculoskeletal system | 687 | 1554 | 359 |
| N00-N99: Diseases of the genitourinary system | 33 | 51 | na |
| O00-O99: Pregnancy, childbirth and the puerperium | 18 | 21 | 235 |
| P00-P96: Certain conditions originating in the perinatal period | na | na | - |
| Q00-Q99: Congenital malformations, deformations and chromosomal abnormalities | na | na | na |
| R00-R99: Symptoms, signs and abnormal clinical laboratory findings | 107 | 224 | 55 |
| S00-T98: Injury, poisoning and certain other consequences of external causes | 183 | 456 | 79 |
| V01-Y98: External causes of morbidity and mortality | na | na | - |
| Z00-Z99: Factors influencing health status and contact with health services | 33 | 74 | 20 |
| Missing diagnosis | 324 | 760 | 317 |

**Supplemental Table 1** Frequencies of ICD-10 main diagnosis categories for SA among those with inpatient or specialized outpatient care during the follow-up

na= less than 10 cases

**Supplemental Table 2** Frequencies of diagnoses of inpatient and specialized outpatient care episodes among individuals with and without sickness absence (SA)

|  | SA | | | | No SA | | | |
| --- | --- | --- | --- | --- | --- | --- | --- | --- |
| ICD-10 main categories for inpatient or specialized outpatient care | Inpatient care (n = 3 217) | | Outpatient care (n= 6 274) | | Inpatient care (n = 25 872) | | Outpatient care (n= 51 669) | |
|  | **n** | **%** | **n** | **%** | **n** | **%** | **n** | **%** |
| A0-A99, B0-B99: Certain infectious and parasitic diseased | 70 | 2 | 79 | 2 | 616 | 2 | 590 | 1 |
| C00-D48, Neoplasms | 168 | 5 | 227 | 4 | 2189 | 9 | 2838 | 7 |
| D50-D89: Diseases of the blood and blood-forming organs | 16 | 1 | 22 | 0 | 181 | 1 | 142 | 0 |
| E00-E90: Endocrine, nutritional and metabolic diseases | 59 | 2 | 88 | 2 | 457 | 2 | 764 | 2 |
| F00-F99: Mental and behavioural disorders | 196 | 6 | 272 | 5 | 1253 | 5 | 1276 | 3 |
| G00-G99: Diseases of the nervous system | 76 | 2 | 137 | 3 | 782 | 3 | 728 | 2 |
| H00-H59: Diseases of the eye and adnexa | 17 | 1 | 266 | 5 | 249 | 1 | 4171 | 10 |
| H60-H95: Diseases of the ear and mastoid process | 30 | 1 | 140 | 3 | 208 | 1 | 1133 | 3 |
| I00-I99: Diseases of the circulatory system | 265 | 8 | 203 | 4 | 4129 | 16 | 2248 | 6 |
| J00-J99: Diseases of the respiratory system | 135 | 4 | 141 | 3 | 1361 | 5 | 1117 | 3 |
| K00-K93: Diseases of the digestive system | 255 | 8 | 223 | 4 | 2148 | 8 | 1871 | 6 |
| L00-L99: Diseases of the skin and subcutaneus tissue | 15 | 5 | 208 | 3 | 166 | 6 | 1974 | 3 |
| M00-M99: Diseases of the musculoskeletal system | 305 | 10 | 767 | 4 | 1953 | 8 | 3776 | 5 |
| N00-N99: Diseases of the genitourinary system | 153 | 5 | 370 | 9 | 1321 | 5 | 2849 | 15 |
| O00-O99: Pregnancy, childbirth and the puerperium | 668 | 21 | 107 | 7 | 2441 | 10 | 360 | 7 |
| P00-P96: Certain conditions originating in the perinatal period | - |  | - |  | - |  | - |  |
| Q00-Q99: Congenital malformations, deformations and chromosomal abnormalities | na | 0 | na | 2 | 65 | 0 | 39 | 1 |
| R00-R99: Symptoms, signs and abnormal clinical laboratory findings | 321 | 10 | 459 | 0 | 2721 | 11 | 3907 | 0 |
| S00-T98: Injury, poisoning and certain other consequences of external causes | 337 | 11 | 638 | 9 | 2617 | 10 | 4502 | 10 |
| Z00-Z99: Factors influencing health status and contact with health services | 101 | 3 | 781 | 12 | 808 | 3 | 6199 | 11 |

na= less than 10 cases

**Supplemental Table 3** Cox proportional hazards regressions (HR) with 95% confidence intervals (CI) for associations between SA diagnoses at ICD-10 main diagnosis categories and in- or outpatient health care

| ICD-10 main categories for SA (no SA as reference) | Inpatient care | | Outpatient care | |
| --- | --- | --- | --- | --- |
|  | **HR** | **95%CI** | **HR** | **95%CI** |
| A0-A99, B0-B99: Certain infectious and parasitic diseased | 4.38 | 2.96, 6.46 | 2.35 | 1.76, 3.15 |
| C00-D48, Neoplasms | 50.61 | 18.58, 137.91 | 26.97 | 10.52, 69.16 |
| D50-D89: Diseases of the blood and blood-forming organs | 6.20 | 2.43, 15.81 | 4.33 | 2.12, 8.84 |
| E00-E90: Endocrine, nutritional and metabolic diseases | 6.39 | 3.08, 13.24 | 4.07 | 2.34, 8.84 |
| F00-F99: Mental and behavioural disorders | 2.28 | 2.02, 2.57 | 1.61 | 1.49, 1.73 |
| G00-G99: Diseases of the nervous system | 3.57 | 1.99, 6.38 | 3.29 | 2.24, 4.84 |
| H00-H59: Diseases of the eye and adnexa | 5.50 | 0.87, 34.93 | 4.29 | 1.19, 15.40 |
| H60-H95: Diseases of the ear and mastoid process | 4.14 | 2.09, 8.19 | 2.66 | 1.55, 4.54 |
| I00-I99: Diseases of the circulatory system | 10.51 | 6.04, 18.30 | 3.41 | 1.88, 6.18 |
| J00-J99: Diseases of the respiratory system | 2.84 | 2.23, 3.60 | 1.80 | 1.53, 2.10 |
| K00-K93: Diseases of the digestive system | 14.91 | 9.60, 23.17 | 7.14 | 4.77, 10.69 |
| L00-L99: Diseases of the skin and subcutaneus tissue | 3.08 | 1.31, 7.22 | 3.11 | 1.85, 5.24 |
| M00-M99: Diseases of the musculoskeletal system | 2.38 | 2.08, 2.71 | 1.83 | 1.68, 1.98 |
| N00-N99: Diseases of the genitourinary system | 9.94 | 4.62, 21.36 | 3.47 | 1.60, 7.53 |
| O00-O99: Pregnancy, childbirth and the puerperium | 2.03 | 1.28, 3.21 | 0.89 | 0.58, 1.36 |
| P00-P96: Certain conditions originating in the perinatal period | - | - | 0.90 | 0.87, 0.93 |
| Q00-Q99: Congenital malformations, deformations and chromosomal abnormalities | - | - | 0.65 | 0.26, 1.63 |
| R00-R99: Symptoms, signs and abnormal clinical laboratory findings | 3.85 | 0.33, 43.99 | 2.45 | 1.92, 3.11 |
| S00-T98: Injury, poisoning and certain other consequences of external causes | 2.92 | 2.25, 3.78 | 2.68 | 2.25, 3.18 |
| V01-Y98: External causes of morbidity and mortality | 3.79 | 0.33, 43.98 | 3.40 | 1.08, 10.75 |
| Z00-Z99: Factors influencing health status and contact with health services | 2.81 | 1.81, 4.36 | 1.96 | 1.54, 2.50 |
| Missing diagnosis | 1.91 | 1.64, 2.22 | 1.37 | 1.25, 1.50 |
